# Supplementary material for: Antifungal Potential of Canarian Plant Extracts against High-Risk Phytopathogens
Source: Plants (Basel). 2022 Nov 5;11(21):2988. doi: 10.3390/plants11212988 (PMC9656886; doi:10.3390/plants11212988)
Supplement: Supplementary file 1 [file plants-11-02988-s001.zip › plants-1995911-supplementary.pdf]

Supporting Information

Antifungal Potential of Canarian Plant Extracts against High-risk Phytopathogens in Crops

Carolina P. Reyes[1], Samuel Rodríguez Sabina [2], Rocío López-Cabeza[1], Cristina G. Montelongo[2], Cristina Giménez[2], Ignacio A. Jiménez[1], Raimundo Cabrera[2] and Isabel L. Bazzochi[1,*]

[1] Instituto Universitario de Bio-Orgánica Antonio González, Departamento de Química Orgánica, Universidad de La Laguna, Avenida Astrofísico Francisco Sánchez 2, 38206 La Laguna, Tenerife, Spain.

[2] Departamento de Botánica, Ecología y Fisiología Vegetal, Facultad de Ciencias, Sección Biología, Universidad de La Laguna, Avenida Astrofísico Francisco Sánchez, 38206 La Laguna, Tenerife, Spain.

* Correspondence: ilopez@ull.edu.es (I.L.B); Tel.: +34922318594

Table of Contents

Table S1. Antifungal effects (% growth inhibition) of plant extracts against *Alternaria alternata*, *Botrytis cinerea* and *Fusarium oxysporum.*

Table S2. Antifungal effects (% growth inhibition) of selected plant fractions against *Alternaria alternata*, *Botrytis cinerea* and *Fusarium oxysporum.*

Figure S1. *In vitro* test-assay of plant extracts against *Alternaria alternate*, *Botrytis cinerea* and *Fusarium oxysporum.*

Table S1. Antifungal effects (% growth inhibition) of plant extracts against *Alternaria alternata, Botrytis cinerea* and *Fusarium oxysporum.*

**Plant extract**	***A. alternata***			***B. cinerea***			***F. oxysporum***		
	1 mg/mL	0.5 mg/mL	0.1 mg/mL	1 mg/mL	0.5 mg/mL	0.1 mg/mL	1 mg/mL	0.5 mg/mL	0.1 mg/mL
C. fusca	NA	ND	ND	28.91 ± 8.49	24.89 ± 6.71	18.94 ± 6.27	NA	ND	ND
A. broussonetii	NA	ND	ND	NA	ND	ND	NA	ND	ND
A. thuscula	23.63 ± 1.48	21.61 ± 5.32	13.34 ± 3.47	12.41 ± 0.55	ND	ND	41.21 ± 1.95	32.33 ± 4.10	19.29 ± 2.92
G. cassinoides	NA	ND	ND	NA	ND	ND	NA	ND	ND
C. symphytifolius	38.10 ± 10.85	18.96 ± 3.73	ND	39.08 ± 3.70	37.98 ± 4.72	19.57 ± 3.32	35.27 ± 4.92	NA	ND
S. canariensis	47.60 ± 0.70	44.61 ± 1.30	27.36 ± 2.54	62.16 ± 4.36	56.23 ± 3.90	35.13 ± 7.83	47.95 ± 1.82	44.76 ± 1.17	33.21 ± 1.93
A. barbujana	NA	ND	ND	NA	ND	ND	NA	ND	ND
L. novocanariensis	NA	ND	ND	NA	ND	ND	16.60 ± 3.57	ND	ND
L. canariensis	NA	ND	ND	NA	ND	ND	NA	ND	ND
R. pinnata	NA	ND	ND	28.27 ± 7.49	NA	ND	25.47 ± 4.38	NA	ND
R. chalepensis	17.08 ± 3.81	ND	ND	NA	ND	ND	44.09 ± 1.63	NA	ND
D. innoxia	37.95 ± 2.40	35.14 ± 4.34	25.75 ± 4.40	52.11 ± 5.22	30.48 ± 5.19	13.28 ± 3.27	20.74 ± 4.40	NA	ND
D. stramonium	NA	ND	ND	NA	ND	ND	NA	ND	ND
N. glauca	NA	ND	ND	NA	ND	ND	NA	ND	ND
S. origanifolia	NA	ND	ND	NA	ND	ND	NA	ND	ND
W. aristata	22.99 ± 7.97	NA	ND	72.22 ± 9.44	52.57 ± 4.96	27.79 ± 7.69	25.39 ± 3.92	13.16 ± 2.09	ND
Methylparaben	100.00 ± 0.00	85.11 ± 8.66	21.67 ± 5.70	100.00 ± 0.00	100.00 ± 0.00	34.13 ± 5.55	100.00 ± 0.00	82.78 ± 5.93	45.92 ± 9.64

Extracts with an inhibition growth higher than 20% at 1 mg/mL were assayed at lower concentrations (0.5 and 0.1 mg/mL). % Growth Inhibition: Means ± standard deviation (SD). NA : not active (% inhibition ≤10); ND : not determined; Methylparaben was used as a positive control.

Table S2. Antifungal effects (% growth inhibition) of selected plant fractions against *Alternaria alternata*, *Botrytis cinerea* and *Fusarium oxysporum.*

**Plant**	**Sample***		***A. alternata***			***B. cinerea***			***F. oxysprorium***	
		1 mg/mL	0.5 mg/mL	0.1 mg/mL	1 mg/mL	0.5 mg/mL	0.1 mg/mL	1 mg/mL	0.5 mg/mL	0.1 mg/mL
A. thuscula	Extract EtOH	23.63 ± 1.48	21.61 ± 5.32	13.34 ± 3.47	12.41 ± 0.55	ND	ND	41.21 ± 1.95	32.33 ± 4.10	19.29 ± 2.92
	A-F1	ND	ND	ND	ND	ND	ND	37.94 ± 5.50	NA	ND
	A-F2	ND	ND	ND	ND	ND	ND	18.68 ± 6.19	ND	ND
	A-F3	ND	ND	ND	ND	ND	ND	NA	ND	ND
C. symphytifolius	Extract EtOH	38.10 ± 10.85	18.96 ± 3.73	ND	39.08 ± 3.70	37.98 ± 4.72	19.57 ± 3.32	35.27 ± 4.92	NA	ND
	C-F1	51.32 ± 4.28	54.46 ± 3.04	45.72 ± 3.60	88.43 ± 5.34	85.80 ± 6.56	69.54 ± 5.37	44.24 ± 1.52	35.21 ± 2.15	27.44 ± 1.99
	C-F2	54.95 ± 2.27	59.72 ± 3.54	36.67 ± 2.21	84.72 ± 3.66	80.54 ± 7.95	69.20 ± 11.46	42.01 ± 2.32	38.48 ± 2.46	26.59 ± 2.40
	C-F3	11.76 ± 1.58	ND	ND	16.77 ± 4.35	ND	ND	36.84 ± 4.62	29.86 ± 2.63	11.62 ± 3.45
S. canariensis	Extract EtOH	47.60 ± 0.70	44.61 ± 1.30	27.36 ± 2.54	62.16 ± 4.36	56.23 ± 3.90	35.13 ± 7.83	47.95 ± 1.82	44.76 ± 1.17	33.21 ± 1.93
	S-F1	59.51 ± 1.92	58.31 ± 1.85	60.55 ± 1.88	86.27 ± 2.88	83.93 ± 4.57	69.14 ± 2.89	65.54 ± 2.20	69.94 ± 4.09	46.85 ± 2.07
	S-F2	57.37 ± 2.29	52.71 ± 3.01	34.64 ± 1.35	78.10 ± 2.51	75.36 ± 5.34	58.54 ± 11.73	56.08 ± 1.76	49.92 ± 2.94	33.04 ± 4.76
	S-F3	NA	ND	ND	NA	ND	ND	NA	ND	ND
R. chalepensis	Extract EtOH	17.08 ± 3.81	ND	ND	NA	ND	ND	44.09 ± 1.63	NA	ND
	R-F1	ND	ND	ND	ND	ND	ND	78.11 ± 1.12	67.96 ± 2.38	12.29 ± 2.08
	R-F2	ND	ND	ND	ND	ND	ND	36.44 ± 1.72	30.05 ± 3.33	13.49 ± 1.61
	R-F3	ND	ND	ND	ND	ND	ND	NA	ND	ND
D. innoxia	Extract EtOH	37.95 ± 2.40	35.14 ± 4.34	25.75 ± 4.40	52.11 ± 5.22	30.48 ± 5.19	13.28 ± 3.27	20.74 ± 4.40	NA	ND
	D-F1	NA	ND	ND	13.85 ± 2.43	ND	ND	ND	ND	ND
	D-F2	23.01 ± 4.58	12.74 ± 3.63	ND	50.28 ± 8.84	32.36 ± 7.90	23.45 ± 5.49	ND	ND	ND
	D-F3	39.36 ± 6.39	15.38 ± 3.84	ND	68.49 ± 7.94	60.99 ± 8.30	27.86 ± 8.37	ND	ND	ND
W. aristata	Extract EtOH	22.99 ± 7.97	NA	ND	72.22 ± 9.44	52.57 ± 4.96	27.79 ± 7.69	25.39 ± 3.92	13.16 ± 2.09	ND
	W-F1	ND	ND	ND	25.56 ± 7.00	NA	ND	ND	ND	ND
	W-F2	ND	ND	ND	51.72 ± 5.06	NA	ND	ND	ND	ND
	W-F3	ND	ND	ND	84.30 ± 4.77	NA	ND	ND	ND	ND

F1: hexanes fraction; F2: ethyl acetate fraction; F3: water fraction. % Growth Inhibition: Means ± standard deviation (SD).
NA: not active (% inhibition ≤10); ND: not determined; Methylparaben was used as a positive control.

(A) (B) (C)

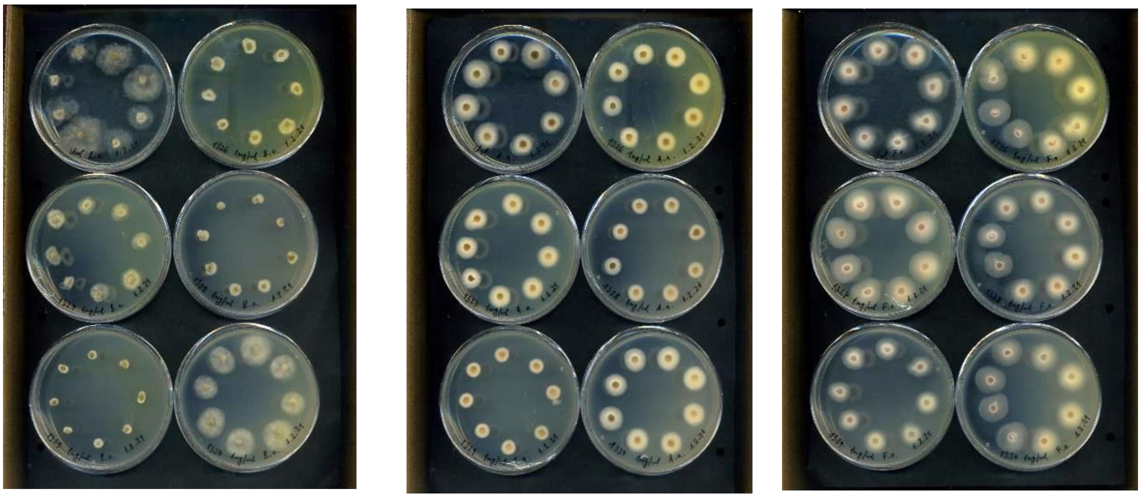

Figure S1. *In vitro* test-assay of plant extracts against *Alternaria alternata* (**A**), *Botrytis cinerea* (**B**) and *Fusarium oxysporum* (**C**).
